# Differential Neural Processing of Social Exclusion and Inclusion in Adolescents with Non-Suicidal Self-Injury and Young Adults with Borderline Personality Disorder

**DOI:** 10.3389/fpsyt.2017.00267

**Published:** 2017-11-29

**Authors:** Rebecca C. Brown, Paul L. Plener, Georg Groen, Dominik Neff, Martina Bonenberger, Birgit Abler

**Affiliations:** ^1^Department of Child and Adolescent Psychiatry/Psychotherapy, University of Ulm, Ulm, Germany; ^2^Department of Psychiatry and Psychotherapy, University of Ulm, Ulm, Germany

**Keywords:** self-injury, non-suicidal self-injury, borderline personality disorder, fMRI, social situations

## Abstract

**Introduction:**

Non-suicidal self-injury (NSSI) is a symptom of borderline personality disorder (BPD). However, NSSI often occurs independently of BPD. Altered neural processing of social exclusion has been shown in adolescents with NSSI and adults with BPD with additional alterations during social inclusion in BPD patients. Aims of this study were to investigate differences in neural processing of social inclusion and exclusion situations between adolescents with NSSI and young adults with BPD and NSSI.

**Methods:**

Using fMRI, neural processing of positive and negative social situations (paradigm: “Cyberball”) was explored. Participants were 14 adolescents with NSSI, but without BPD (M_age_ = 15.4; SD = 1.9), 15 adults with BPD and NSSI (M_age_ = 23.3; SD = 4.1), as well as 15 healthy adolescents (M_age_ = 14.5; SD = 1.7), and 16 healthy adults (M_age_ = 23.2; SD = 4.4).

**Results:**

Behavioral results showed enhanced feelings of social exclusion in both patient groups as compared to healthy controls but only the NSSI group showed enhanced activation during social exclusion versus inclusion compared to the other groups. While both NSSI and BPD groups showed enhanced activation in the ventral anterior cingulate cortex during social exclusion as compared to their age-matched controls, enhanced activation during social inclusion as compared to a passive watching condition was mainly observed in the BPD group in the dorsolateral and dorsomedial prefrontal cortex, and the anterior insula.

**Discussion:**

While neural processing of social exclusion was pronounced in adolescents with NSSI, BPD patients also showed increased activity in a *per se* positive social situation. These results might point toward a higher responsiveness to social exclusion in adolescents with NSSI, which might then develop into a generalized increased sensitivity to all kinds of social situations in adults with BPD.

## Introduction

Non-suicidal self-injury (NSSI), defined as the intentional and direct damage of body tissue without suicidal intent, is described as a prominent and maybe the most noticeable symptom of borderline personality disorder (BPD) However, lifetime prevalence rates of BPD range around 2.7% in the general population (1), while around 18% of adolescents report to have engaged in NSSI at least once, and repetitive NSSI is seen in around 4 and 7% of adolescents (2, 3). From an epidemiological perspective, these numbers suggest that NSSI also occurs independently of BPD in a large number of adolescents, which is why NSSI has been suggested as a distinct psychiatric disorder in the most current version of the Diagnostic and Statistical Manual [DSM-5 (4)] as condition for further study.

Still, there are commonalities between both entities. According to the DSM-5, BPD is characterized by impairments of interpersonal functioning, which can be associated to “interpersonal hypersensitivity (i.e., prone to feel slighted or insulted)” and “perceptions of others selectively biased toward negative attributes or vulnerabilities” (4). In laboratory studies, adults with BPD have repeatedly shown an increased sensitivity for social exclusion in comparison to healthy controls (5, 6). Similarly, NSSI has also been linked with impaired social interactions. For example, bullying has been shown to be a risk factor in longitudinal studies (7, 8) and patients with NSSI show elevated feelings of loneliness, even in comparison to clinical controls (9). Consequently, while BPD and NSSI do not match well in prevalence rates, sensitivity to social exclusion may play a role in both syndromes.

Investigations of the neural correlates of social exclusion [evoked by the paradigm “Cyberball” (10)] have shown increased activation in various brain regions, including the anterior cingulate cortex (ACC), anterior insula/ventrolateral prefrontal cortex (vlPFC), medial prefrontal cortex (mPFC), dorsolateral prefrontal cortex (dlPFC), and ventral striatum (11–14). While dorsal ACC, ventral striatum and anterior insula/vlPFC have been conceptualized as a network processing salience, dorsolateral and dorsomedial prefrontal regions, together with lateral parietal areas are conceptualized as a central executive network (15). The salience system has been suggested as the cerebral network where sensory data with behavioral relevance are integrated with visceral, autonomic, and hedonic signals while the central executive system might operate on the identified salience. Such operations, for example, comprise directing attention to pertinent stimuli when behavioral choices are weighed against shifting conditions, background homeostatic demands, and context (16). In adult patients with BPD, altered activation of the dorsal ACC and the dorsal mPFC was shown to relate to social exclusion, but interestingly also to social inclusion as compared to healthy controls (17). The authors suggested that this might point toward a generalized “hypermentalizing” or alarm reaction in patients with BPD in both negative and positive social situations. In a study investigating depressed adolescents with and without repetitive NSSI, we found that activation in the mPFC and vlPFC was not only different from healthy controls but also modulated by the presence or absence of NSSI (18). However, no differences during social inclusion were found across those three groups. We took these results as hint for a potentially distinct neurobiology of NSSI compared to BPD.

Given this discrepancy between patient populations despite the overlap in the clinical presentation of BPD and NSSI, we aimed at a better understanding of commonalities and differences on a neurobiological level. We further investigated this issue by recruiting patients with ongoing NSSI as a symptom with and without a diagnosis of BPD. As NSSI without BPD is most frequent during adolescence, while diagnoses of BPD are often not made before early adulthood, we recruited subjects from post puberty to early adulthood. To control for effects of age, we investigated matched healthy controls. In accord with previous finding using the same task, we hypothesized that both patient groups would show increased activation in relevant brain areas during social exclusion as compared to healthy controls. We also conjectured that only those NSSI participants with an additional BPD diagnosis would also show altered activation during social inclusion.

## Materials and Methods

### Participants

In total, 59 participants were recruited for this study. Of those, 27 were patients with ongoing NSSI (at least five times within the past year, meeting proposed DSM-5 criteria for NSSI), of which 13 were adolescents without a diagnosis of BPD [“NSSI group,” mean age = 15.5 years (SD = 2.0)], and 14 were young adults with BPD [“BPD group,” mean age = 23.6 (SD = 4.1)]. All patients were also diagnosed with major depression. The other 32 individuals were grouped into two healthy control groups with 15 adolescents [“HCG-Y,” mean age = 14.5 years (SD = 1.7)] and 17 young adults [“HCG-A,” mean age = 23.2 years (SD = 4.4)] (see Table 1). Participants were recruited from inpatient and outpatient units of the University Hospital for Child and Adolescent Psychiatry and Psychotherapy and the Department of Psychiatry and Psychotherapy of Ulm University, Germany, and from a private medical practice for child and adolescent psychiatry in Ulm. Two of all participants were left handed, 14 reported to smoke cigarettes regularly (smoking was prohibited at least 2 h before fMRI), and one of the participants reported excessive use of alcohol, as assessed by corresponding sections of the K-SADS-PL for adolescents and the Structured Clinical Interview for DSM-IV, axis I (SCID-I) for adults (descriptions see below). Current medical disorders, epilepsy, substance use disorders, and psychotic disorders were exclusion criteria. All adolescent participants had reached puberty and all female participants were scanned during the luteal phase of the menstrual cycle or after at least 14 days of continuous intake of oral contraception.

**Table 1:** Characteristics of participants.

	NSSI	HCG-Y	Chi^2^ (*p*)	BPD	HCG-A	Chi^2^ (*p*)
*N*	13	15		14	17	
Gender (female)	76.9%	80.0%	0.84 (*p* > 0.05)	100%	100%	–

	**M (SD)**	**M (SD)**	***T* (*p*)**	**M (SD)**	**M (SD)**	***T* (*p*)**

Age	15.5 (2.0)	14.5 (1.7)	1.4 (*p* > 0.05)	23.6 (4.1)	23.2 (4.4)	0.3 (*p* > 0.05)
**Medication**						
Antidepressants	2	–		12	–	
Mood stabilizers	–	–		1	–	
**Axis 1 disorders**						
Major depression	13	–		14	–	
Hyperkinetic disorder	3	–		–	–	
Eating disorder	2	–		1	–	
Anxiety disorder	2	–		2	–	
PTSD	–	–		7	–	

	**M (SD)**	**M (SD)**		**M (SD)**	**M (SD)**	

**Incidents NSSI**						
Lifetime	128.8 (277.4)	–		308.5 (450.5)	–	
12 months	31.2 (33.3)	–		34.2 (36.7)	–	
BDI-II score	20.8 (12.2)	2.7 (3.5)		41.7 (12.6)	3.2 (3.5)	

*BDI-II, Beck’s Depression Inventory, 2nd edition*.

Patients and their respective controls were matched according to gender and age (see Table 1). Antidepressant medication was not interrupted due to ongoing treatment of patients. However, in order to assure steady-state conditions, medication was held stable for at least 2 weeks prior to fMRI scanning and no sedative *pro re nata* medication was allowed prior to scanning. Of the adult patients, 12 took antidepressant medication of various kinds, of these, one patient was additionally treated with lithium. Two adolescent patients received antidepressants. Treatment with antipsychotic medication was an exclusion criterion. The study was approved by the Institutional Review Board of Ulm University, Ulm, Germany. Written informed consent was obtained from legal guardians (where applicable) and participants. All procedures were performed according to the Declaration of Helsinki.

### Psychometric Measurements

According to the age of participants, different tools were used for the assessment of psychiatric symptoms of participants. The German version of the clinical interview *Schedule for Affective Disorders and Schizophrenia for School-Age-Children-Present and Lifetime* (K-SADS-PL) for DSM-IV diagnoses (19) was used to assess adolescents of the NSSI and healthy control groups. In the adult groups, the *Structured Clinical Interview for DSM-IV, axis I (SCID-I)* was used for assessment. The semi-structured *Self-injurious thoughts and behaviors interview* [SITBI (20), German version (21)] was used in all groups to obtain detailed information about participants’ NSSI (present and lifetime). In order to assess current depressive symptoms, the *Beck Depression Inventory, second edition* [BDI-II (22); German version (23)] was applied in all four groups.

General sensitivity for social exclusion was assessed by the Hurt-Feelings-Scale [HFS (24)]. The HFS consists of eight questions concerning the sensitivity in social situations (i.e., “I rarely feel hurt by what other people say or do to me”). Each question is rated on a 5-point Likert-scale. A higher score corresponds to a higher sensitivity to social exclusion. The 20-item *Need-Threat-Scale* (25) was used for measuring the extent of feeling socially excluded after “Cyberball.” Each item was rated on a scale from 1 (not at all) to 5 (very much). Total scores were calculated by dividing the final result by 20. Therefore, reaction to “Cyberball” can range from 1 (no distress) to 5 (very high distress). The same rating has already been applied in previous fMRI studies using Cyberball (14, 26).

### Experimental Task

The well-established task “Cyberball” (10) was used in order to examine processing of positive (inclusion) and negative (exclusion) social situations. Participants believed to be playing a virtual ball-tossing game with other participants in another room. In reality, all actions were pre-programmed and the other players did not exist [for details, see Ref. (10, 13)]. Participants were represented by a hand at the bottom part of the screen, while the other players were represented by animated comic figures. All participants played three rounds of “Cyberball,” with every round lasting around 2 min (60 throws). In the first round (“Passive”), participants were told their character was controlled by a computer and their task was to watch the game. In the second round (“Inclusion”), participants received the ball randomly in one-third of all throws. The third round (“Exclusion”) started with 10 throws of “Inclusion,” but thereafter participants did no longer receive the ball for the remaining 50 throws. Information about the necessity of deception in this experiment and the real nature of the game was given in a debriefing session in verbal and written form.

### Functional Data Acquisition

A 3.0-T MAGNETOM Allegra Scanner (Siemens, Erlangen, Germany) was used to obtain fMRI data. A T2*-sensitive gradient echo sequence was used for functional imaging, with an echo time (TE) of 33 ms, a flip angle of 90°, a field of view of 230 mm, and a slice thickness of 2.5 mm with an interslice gap of 0.5 mm. At a repetition time (TR) of 2,000 ms, 35 transversal slices were recorded with an image size of 64 × 64 pixels during the Cyberball task. Anatomical high-resolution T1-weighted images (1 mm × 1 mm × 1 mm voxels) were acquired (band-width = 130 Hz/Pixel, TR = 2500 ms, TI = 1.1 s, TE = 4.57 ms, flip angle = 12°) for reasons of co-registration and normalization into standardized stereotactic space.

### Data Analysis

#### Behavioral Data

Statistical differences in ratings of the HFS, Need-Threat-Scale, and depression scores were calculated in SPSS (IBM SPSS Statistics, version 21), using univariate analyses of variance (ANOVAs) and *post hoc* two-tailed *t*-tests.

#### fMRI-Data

The Statistical Parametric Mapping Package 8 (SPM 8, Wellcome Trust Centre for Neuroimaging, London, UK) was used for image data pre-processing and statistical analyses. Head movement and slice acquisition delay was controlled by realignment and slice timing during pre-processing. Images were normalized to the standard MNI-template (Montreal Neurological Institute). Spatial smoothing was conducted using an 8 mm full width at half maximum Gaussian kernel. Low-frequency drifts were removed *via* high pass filtering, and an AR(1) model was used to account for intrinsic autocorrelations.

For individual first-level analyses, a general linear model was used to estimate the variance of voxels for each condition. The Cyberball task was modeled as three separate blocks of conditions (passive watching, inclusion, and exclusion) as has been a common procedure in previous studies (13, 27). Regressors representing the six motion parameters were integrated into the design matrix.

For second-level group analysis, we set up a 4 × 3 flexible factorial ANOVA model with factors “group” (NSSI, HCG-Y, BPD, HCG-A) and “condition,” and included first-level contrast images for the three ball-tossing conditions for each of the four groups. We calculated an *F*-Test to examine the main effect of “condition” (passive, inclusion, exclusion) across all groups. We also calculated an omnibus *F*-Test for the interaction of group and condition. Following Lieberman and Cunningham (28) and Domsalla et al. (17), we only report voxels that met an uncorrected threshold of *p* < 0.001 and were part of a cluster larger than 10 contiguously significant voxels. According to previous studies with the Cyberball experiment [e.g., Ref. (13, 14)], the contrasts of interest were exclusion > inclusion and according to the finding of Domsalla et al. (17) in borderline patients also the contrast inclusion > passive viewing. Planned *post hoc t*-tests for those two contrasts were then conducted using SPSS for Windows to explore differences between groups for peak activations identified by the omnibus *F*-Test. The nominal level of significance was set at *p* < 0.05 for *post hoc* testing.

*Post hoc* results were only considered meaningful in cases where differences between patient groups were also seen when comparing patients with their age-matched controls. If effects comparing the two patient groups were also seen in comparisons of the two age-different control groups, between-patient group results were discarded as mere age effects. Coordinates in text and tables are MNI coordinates.

## Results

### Behavioral Data

Ratings of the HFS differed significantly between groups (*F* = 11.74, *p* < 0.001). Participants of the BPD group (M_BPD_ = 27.0, SD_BPD_ = 3.59) showed significantly higher sensitivity to social rejection (trait) compared to adult healthy controls (M_HCG_Adult_ = 17.79, SD_HCG_Adult_ = 4.85; *p* < 0.001, *T* = 5.29). Participants of the NSSI group (M_NSSI_ = 21.8, SD_NSSI_ = 6.42) showed higher scoring than adolescent healthy controls (M_HCG_Youth_ = 16.2, SD_HCG_Youth_ = 4.5; *p* < 0.05, *T* = 2.62). Also, adults with BPD scored significantly higher than adolescents with NSSI (*p* < 0.05, *T* = 2.76), while there were no differences between healthy adults and healthy adolescents (*p* > 0.05, *T* = 0.91).

Ratings of social exclusion directly after Cyberball, as measured by the NTS, differed significantly between groups (*F* = 7.74, *p* < 0.001). Participants in the BPD group (M = 3.51, SD = 0.31) rated feelings of social exclusion significantly higher than participants in the NSSI group (M = 2.93, SD = 0.84; *p* < 0.05, *T* = 2.42), and healthy controls (M = 2.55, SD = 0.50; *p* < 0.001, *T* = 6.09). There were no significant differences between the NSSI group and age-matched healthy controls (M = 2.55, SD = 0.69, *p* = 0.14, *T* = 1.52). Also, both healthy control groups did not differ on social exclusion ratings (*p* > 0.05, *T* = 0.003).

### Functional Imaging Data

#### Main Effect of Task

Across all four groups, the main effect of task (passive, inclusion, exclusion) confirmed activation of brain regions previously described during Cyberball. These regions included the vlPFC (right: *x*/*y*/*z* = 30/28/−12, *z* = 4.62, *p* < 0.001 and left: *x*/*y*/*z* = −30/20/−8, *z* = 3.85, *p* < 0.001), pregenual ACC (*x*/*y*/*z* = −6/48/−4, *z* = 3.63, *p* < 0.001), dorsal ACC (*x*/*y*/*z* = 2/12/30, *z* = 4.39, *p* < 0.001) and ventral striatum (right: *x*/*y*/*z* = 12/10/−4, *z* = 5.50, *p* < 0.001 and left: *x*/*y*/*z* = −10/10/−2, *z* = 5.23, *p* < 0.001), but also the dlPFC (*x*/*y*/*z* = −24/−4/54, *z* = 4.73, *p* < 0.001).

#### Interaction Effect of Group by Condition

An omnibus *F*-test on the interaction of factors group and condition showed significant effects in dorsolateral and dorsomedial prefrontal regions, the anterior insula, and the putamen (see Table 2).

**Table 2:** Interaction effect of group and condition.

	L/R	BA	*x*/*y*/*z*	*k*	*Z*	*p*
Dorsolateral prefrontal cortex	L	9	−44/34/28	12	3.35	<0.001
Dorsomedial prefrontal cortex	L	8	−18/22/52	44	3.91	<0.001
Putamen	L	49	−18/16/−4	67	4.27	<0.001
Anterior insula	L	13	−28/16/16	58	3.22	<0.001
Premotor cortex	R	6	36/0/40	21	3.35	<0.001
	L	6	−26/−2/52	64	4.65	<0.001

*BA, Brodmann’s area*.

#### *Post Hoc* Analyses Regarding the Contrast Exclusion > Inclusion

**Figure 1:**
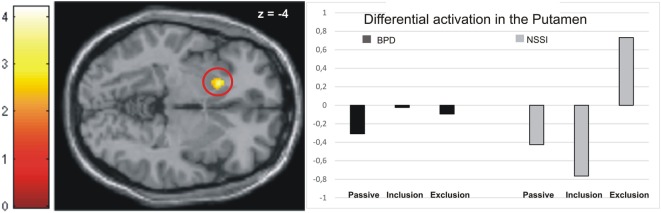
Activation in the putamen in the non-suicidal self-injury (NSSI) and borderline personality disorder (BPD) groups across all three conditions.

Activation in the peak-voxels identified by the omnibus *F*-test on the interaction of factors group by conditions was then analyzed for the difference of exclusion versus inclusion. Univariate ANOVAs revealed a significant between-group difference in the putamen (−18/16/−4: *F* = 4.82, *p* < 0.01), but not in the other brain regions tabulated in Table 2. *Post hoc t*-tests further revealed significant differences between all groups. Specifically, the NSSI group showed enhanced activation as compared to the BPD (*t* = 2.81, *p* < 0.01, see Figure 1) and the matched HCG-Y group (*t* = 2.34, *p* < 0.05), while the HCG-Y group showed lower activation than the HCG-A group (*t* = −2.52, *p* < 0.01), thus ruling out a mere effect of age for the significant difference between both patient groups.

#### *Post Hoc* Analyses of the Contrast Inclusion > Passive Watching

**Figure 2:**
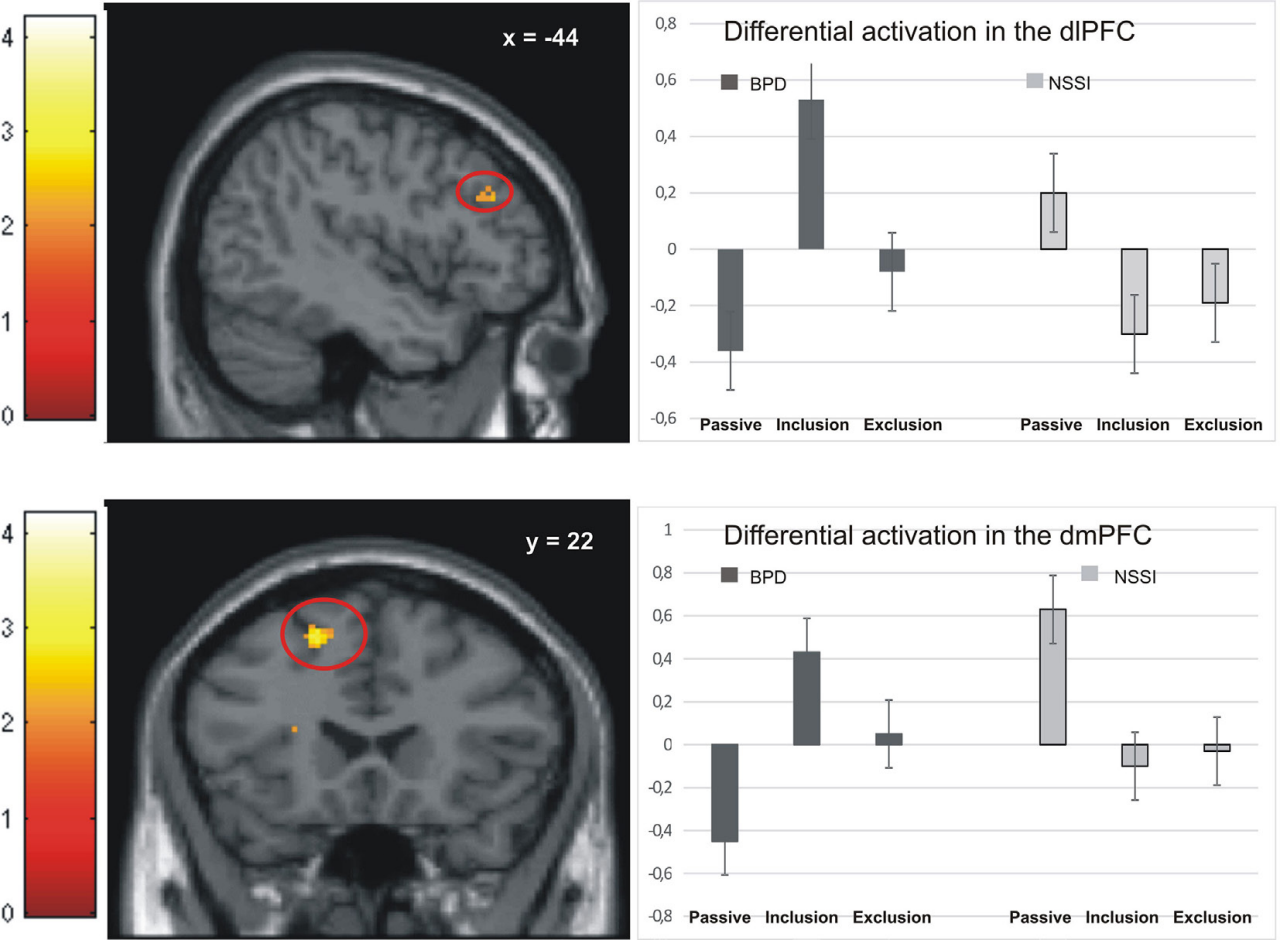
Activation in the dorsolateral prefrontal cortex (dlPFC) and dorsomedial prefrontal cortex (dmPFC) in the non-suicidal self-injury (NSSI) and borderline personality disorder (BPD) groups across all three conditions.

Activation in the peak-voxels identified in the omnibus *F*-test on the interaction of factor group and condition was analyzed for the difference inclusion versus passive watching. Univariate ANOVAs revealed significant between-group differences in the dlPFC (−44/34/28: *F* = 4.77, *p* < 0.01), the premotor cortex (36/0/44: *F* = 4.39, *p* < 0.01), the dorsomedial prefrontal cortex (dmPFC; −18/22/55: *F* = 4.90, *p* < 0.01), and the anterior insula (−28/16/16: *F* = 6.11, *p* < 0.001). *Post hoc t*-tests revealed significant differences between the NSSI and the BPD groups in all regions, with greater activation in BPD. In all regions, significantly enhanced activation in the BPD was also seen when compared to the age-matched HCG-A group, while there was no enhanced activation in the NSSI group relative to their age-matched HCG-Y group. Comparisons of control groups confirmed that the effects of BPD > NSSI was not a mere effect of age (see Table 3), since there was either no group difference between younger and older healthy controls or the difference was in favor of the younger group (see premotor cortex in Table 3) that is in the opposite “age direction” relative to the results of comparing older BPD patients against younger NSSI patients. Pronounced activation during inclusion in the dlPFC and dmPFC in the BPD group can be seen in detail in Figure 2.

**Table 3:** Significant differences between groups regarding the contrast inclusion > passive watching.

Group	*t*	*p*
**Dorsolateral prefrontal cortex (−44/34/28)**		
BPD > NSSI	2.43	<0.05
BPD > HCG_A	3.54	<0.01
HCG_A > HCG_Y	−2.33	>0.05

**Premotor cortex (36/0/40)**		
BPD > NSSI	2.29	<0.05
BPD > HCG_A	3.13	<0.01
HCG_A > HCG_Y	−2.70	<0.05

**Dorsomedial prefrontal cortex (−18/22/55)**		
BPD > NSSI	3.66	<0.01
BPD > HCG_A	2.99	<0.01
HCG_A > HCG_Y	−1.62	>0.05

**Anterior insula (−28/16/16)**		
BPD > NSSI	4.01	<0.01
BPD > HCG_A	3.22	<0.01
HCG_A > HCG_Y	−0.51	>0.05

#### *Post Hoc* Analyses of the Contrast Exclusion > Passive Watching

Regarding the contrast exclusion > passive watching, univariate ANOVAs showed significant between-group differences in the putamen (−18/16/−4: *F* = 4.95, *p* < 0.01), the premotor cortex (36/0/40: *F* = 6.19, *p* < 0.01), and the dmPFC (−18/22/55: *F* = 2.79, *p* < 0.05). *Post hoc t*-tests revealed significantly higher activation in the premotor cortex (*T* = 2.19, *p* < 0.05) and the dmPFC (*T* = 2.49, *p* < 0.05) in the BPD group than the NSSI group, while marginally higher activation in the putamen was found in the NSSI group than the BPD group, but not on a significant level (*T* = −2.00, *p* = 0.06, see Figure 1). Again, these effects were not explained by age, as there were no significant differences between the healthy control groups regarding activation in the dmPFC, and significant differences in the putamen (HCG_Y > HCG_A: *T* = −2.92, *p* < 0.01) and the premotor cortex (HCG_A > HCG_Y: *T* = −4.11, *p* < 0.001) were the opposite direction of age as the patients’ groups effects. As compared to their respective healthy control groups, participants in the NSSI group showed significantly higher levels of activation in the putamen (*T* = 3.66, *p* < 0.01), while participants in the BPD group showed significantly higher activation in the premotor cortex (*T* = 2.89, *p* < 0.01) and the dmPFC (*T* = 2.55, *p* < 0.05).

### Common Effect of Social Exclusion in Both Patient Groups

After investigating differences between patient groups, we calculated a conjunction analysis to explore whether core regions of the network involved during Cyberball were equally active in both patient groups relative to their healthy control groups. The conjunction analysis for the contrast exclusion > inclusion for both patient groups compared to their respective control groups (BPD > HCG_A in conjunction with NSSI > HCG_Y) showed common activation only in the ventral ACC (*x*/*y*/*z* = 12/48/8) down to a lenient threshold of *p* < 0.05 chosen to avoid type-II errors. For the contrast inclusion > passive viewing, no common activations were found.

### Correlation of Behavioral and Imaging Data

We investigated correlations of the HFS with the three main regions found to differ significantly between the two patient groups during social inclusion: dlPFC, dmPFC, and the anterior insula.

Regarding activation in the dmPFC, we found a significant positive correlation with the HFS (*r* = 0.83, *p* < 0.001) in the NSSI group, i.e., those NSSI patients that came closer to BPD with regard to HFS (higher values) showed also the highest brain activation, closest to BPD. No significant correlation was found in the BPD group (HFS: *r* = −0.18, *p* > 0.05), however. Correlations seen in the NSSI group were still evident when the correlation coefficient was calculated across both groups of patients together (HFS: *r* = 0.66, *p* < 0.001). One outlier in the BPD group was removed for calculations.

No correlations were found for the dlPFC or the anterior insula.

## Discussion

This study is the first to compare adolescents with ongoing NSSI without BPD diagnosis against young adults with ongoing NSSI and BPD diagnosis regarding their neural processing of social situations experimentally operationalized by the Cyberball paradigm during fMRI. While both patient groups showed significantly higher ratings on the HFS relative to their age-matched healthy control groups as an index of greater general sensitivity for social exclusion, task-specific ratings of distress experienced from the social exclusion condition of the Cyberball paradigm were significantly higher only in BPD patients relative to controls, but not in NSSI patients. Furthermore, in both rating scales, BPD patients scored higher than patients with NSSI without BPD diagnosis [see also Ref. (5, 6)].

Functionally, the contrast of social exclusion versus inclusion was significant only in the putamen of the NSSI group relative to age-matched controls and BPD patients. At the predefined level of significance, this contrast did not reveal any significant differences in the height of neural activations between BPD patients and healthy controls. Only when the level of significance was lowered for exploratory reasons, both patient groups showed a similarity in comparison to their age-matched healthy controls with relatively increased activation in the ventral ACC for the contrast of social exclusion minus inclusion. This area has previously been reported in two other studies on social exclusion (14, 29) and has functionally been related to the effect of negative social feedback (30). When contrasting social inclusion against passive viewing, a rather consistent pattern of higher activation in the BPD group compared to NSSI patients emerging in the dorsomedial, and dlPFC, and the left anterior insula was found.

Higher activation of the putamen during the exclusion relative to the inclusion and the passive watching condition was observed in the NSSI group only. This was true despite the adolescent patients’ ratings of their general sensitivity to social rejection being significantly lower than those of the BPD group. As the putamen is part of the salience-network (16), this may point toward increased neural reactivity to behaviorally relevant stimuli in adolescents with NSSI. Hyperactivation of nodes of the salience-network may be related to the perception of inconsistencies or norm violation. This may be interpreted in a way that the younger patient group may have processed the negative social feedback more intensely than healthy controls and social exclusion may have been more salient than inclusion. These findings are in line with studies reporting bullying, as severe form of social exclusion, to be a risk factor for NSSI in adolescence (7, 8). However, in the BPD group, putamen activation was indifferent across all three Cyberball conditions. Therefore, this finding should be interpreted with caution and future studies are needed for validation.

Similar to a study comparing patients with BPD to healthy controls (17), we found enhanced activation in the dmPFC in BPD patients during both the inclusion and exclusion conditions. This finding might add to the notion of generalized “hypermentalization” of BPD patients during social exclusion, as proposed by Domsalla and colleagues.

Also in line with Domsalla et al. (17), we found particularly strong neural activation to social inclusion in dorsolateral and dorsomedial prefrontal brain regions in BPD, which was not seen in the NSSI group (Figure 2). To explain this result and the additional finding of self-reports of increased feelings of being excluded and higher trait-sensitivity for exclusion in BPD patients, Domsalla et al. (17) proposed Festinger’s theory of cognitive dissonance (31). For BPD patients, social exclusion could be the situation they anticipate as a result of regularly biased processing in real life. The experimental condition of social inclusion could, therefore, be more unexpected for BPD patients who may then change their subjective perception to match expectations and to stabilize negative beliefs. Increased prefrontal activation as found by Domsalla et al. (17) and in our present study may represent a correlate of the effort to resolve this cognitive dissonance. As suggested by Etkin et al. (32) increased dorsolateral and dorsomedial prefrontal activation could be interpreted as a correlate of enhanced conflict evaluation, while increased salience-network activation as observed in adolescents with NSSI could represent enhanced conflict perception.

The differences between adolescents with NSSI and young adults with BPD (+NSSI) are interesting, since NSSI is one of the core symptoms of BPD. As found in previous fMRI studies (33, 34), pain (and especially tissue damage) seems to have a soothing effect in BPD patients after stress induction on a psychophysiological level and regarding neuronal processing. It was shown that the experience of painful stimuli led to increased connectivity between prefrontal and limbic regions, thus resulting in increased inhibition of limbic areas, and especially the amygdala (33, 34). The Biosocial Development Model of BPD (35) describes a vulnerability early in life, which is initially expressed by elevated impulsivity and later by increased emotional sensitivity. In this model, NSSI as a symptom could be an early indicator for the development of BPD in combination with increased sensitivity to social rejection as a second factor. NSSI and BPD might, therefore, represent a developmental continuum, starting with an increased sensitivity to social exclusion in adolescence, which generalizes to neutral or even positive social situations, thereby increasing symptom load of BPD and severity of this disorder. Correlations of behavioral results with fMRI data in this study can be seen to support this theory. The observation that those NSSI patients, who came closer to the BPD group behaviorally, did so also in terms of neurobiological effects, may support the idea that NSSI in adolescents and BPD in young adults can be seen as two expressions of a continuum. Certainly, longitudinal studies will be necessary to verify this developmental perspective. Longitudinal studies could also substantiate the knowledge whether adolescents with NSSI, who were particularly sensitive to social exclusion in this study, would be at a marked greater risk to develop BPD later on in life and would also develop a still more generalized sensitivity to social situations. From a clinical point of view, understanding why some adolescents with NSSI go on to develop the full symptomatology of BPD, while others do not, is key in tailoring interventions to patients’ needs.

When interpreting the results of this study, the following limitations have to be taken into consideration: the sample sizes of the individual groups included in this study were rather small. Therefore, significant differences may have remained undetected and results cannot be generalized easily. Replication of these findings in larger samples is, therefore, crucial. Ideally, patients with and without a diagnosis of BPD would have been of the same age. However, NSSI without concomitant BPD is very rare in young adults, while a safe diagnosis of developing BPD is comparatively infrequent in adolescents. We tried to overcome this issue by age-matched control groups. Still, a direct comparison between the two groups would have been more ideal but was not possible due to the age difference which is almost impossible to avoid. Furthermore, participants of the BPD group showed significantly higher scores of depression and were more often diagnosed with PTSD than adolescents of the NSSI group. While it is true that all of the participants in the NSSI and the BPD groups had clinically relevant levels of depression, we were able to show in a previous study of matched controls with depression but without NSSI that the effects of social exclusion were specific to NSSI, not to depression or psychiatric impairment in general (18). However, to increase validity of our findings, future replications with studies including clinical controls are necessary. Patients with BPD in this study received a larger proportion of antidepressant medication. Antidepressant medication could, therefore, have been the reason why, for example, the hyperactivation in the putamen was only seen in the NSSI group, but not in the BPD group. Therefore, this result has to be interpreted with caution. Moreover, future studies should evaluate feelings of being excluded separately for the exclusion, inclusion and control conditions as described in Ref. (6, 17). Other factors that may have influenced our results and could be interesting for future research are dissociative states during social exclusion, and differences in psychophysiological reactions.

Strengths of this study were gender- and age-matched healthy controls, by which possible age effects could be controlled for, careful psychological evaluation and the use of a robust paradigm to measure effects of social inclusion and exclusion.

In summary, the present study points toward a distinct neurobiological signature of NSSI in adolescence as compared to BPD in young adulthood. Developmental processes might be involved in a generalized sensitivity to social situations, starting with enhanced sensitivity to social exclusion in adolescence, which is correlated with NSSI and may then expand to neutral and even positive social situations with increasing BPD symptomatology. Future longitudinal studies would be necessary to elucidate this possible developmental pathway.

## Ethics Statement

The study was approved by the Institutional Review Board of Ulm University, Ulm, Germany. Written informed consent was obtained from legal guardians (where applicable) and all participants. All procedures were performed according to the Declaration of Helsinki.

## Author Contributions

RB was involved in designing the study, recruited participants, conducted fMRI scans and psychological interviews, analyzed data, calculated results, and was closely involved in drafting and revising the manuscript. PP and GG gave statistical, methodological, and content advise and revised the manuscript. DN and MB recruited participants, conducted fMRI scans, analyzed data, and revised the manuscript. BA was involved in deigning the study, analyzed data, calculated results, and was closely involved in drafting and revising the manuscript.

## Conflict of Interest Statement

All authors declare no conflict of interest with the potential to bias the work. PP has received research funding from the German federal ministry of research and education, the German federal agency for drugs and medical products, the German research foundation, the Baden-Wuerttemberg Foundation, Lundbeck pharmaceuticals, and the Volkswagen Foundation.
